# Developing and evaluating an intelligent integration system for older adult care and health based on the sport-medicine-tourism model

**DOI:** 10.3389/fpubh.2025.1523893

**Published:** 2025-07-11

**Authors:** Xiaoxue Ma, Xiaofei Fang, Shuqian Liu, Xueqing Wang, Ping Wang, Fugao Jiang

**Affiliations:** ^1^College of Physical Education and Sport Science, Qufu Normal University, Qufu, China; ^2^Faculty of Psychology, Shandong Normal University, Jinan, China; ^3^School of Information Engineering, Shandong Vocational and Technical University of International Studies, Rizhao, China

**Keywords:** intelligent older adult care system, SMT integration, digital aging services, actor-network theory, system evaluation

## Abstract

This study develops a comprehensive intelligent integration system for older adult care and health by combining resources from sport, medicine, and tourism. The research aims to address the dual challenges of population aging and the transformation of older adult care services in the digital era. The study follows a five-stage research framework: theoretical construction, user needs identification, system design, prototype modeling, and empirical evaluation. A 12-month quasi-experimental study was conducted with 300 older adult participants from Jinan, Qingdao, and Rizhao in China, utilizing both control and experimental groups. Physical health, psychological well-being, social participation, and quality of life were measured using standardized scales (GDS-15, SWLS, WHOQOL-BREF), while system performance was assessed using the Analytic Hierarchy Process and fuzzy comprehensive evaluation. The findings demonstrate significant improvements in the experimental group across all health dimensions (*p* < 0.05), and the system was rated as “Good” in functional utility, service quality, and user satisfaction. However, deficiencies in tourism service comfort remain and require further optimization. This study contributes a novel SMT-based integration model supported by STS theory and actor-network modeling, offering theoretical insight and empirical evidence for the design of intelligent older adult care systems in aging societies.

## 1 Introduction

Human society has entered an era marked by digital intelligence competition, in which emerging intelligent technologies have become critical engines for transforming the health and wellness sector. During China’s 14th Five-Year Plan period, addressing population aging has been elevated to a national strategic priority, with scientific and technological innovation—as well as the cultivation of new forms of productive capacity—identified as essential pathways to overcoming longstanding resource shortages and service inefficiencies in conventional older adult care systems (1, 2). Within this evolving landscape, sports-centered wellness services—recognized as effective vehicles for enhancing physical health, psychological well-being, and social inclusion among older adults—are undergoing a structural transformation toward greater integration, intelligence, and systematization (3). Among these developments, the integrated “Sports-Medicine-Tourism” (SMT) model has garnered increasing academic attention as a promising framework for meeting the diverse and layered needs of an aging population. By combining resources from sports, medical services, and tourism sectors, the SMT model establishes cross-sectoral collaboration to facilitate need-responsive health promotion and quality-of-life improvement (4).

Previous research has addressed several dimensions of this interdisciplinary model, including coordination mechanisms in sports-medicine-wellness systems (5), intelligent sports technology applications (6), and hybrid service models that merge medical care with physical activity (7). Additionally, studies have provided empirical evidence on the positive impact of embedding Internet of Things (IoT) and artificial intelligence (AI) technologies within rehabilitation medicine and wellness tourism contexts (8–10). However, systemic challenges remain. Current literature lacks comprehensive top-level system designs that fully integrate the triadic components of SMT. At the level of intelligent operational mechanisms, pathways for service chain optimization via technological empowerment have yet to be firmly established. Furthermore, the seamless linkage of multi-scenario services and efficient allocation of cross-sectoral resources continues to be difficult in practical implementation. Most notably, the field still lacks holistic design frameworks tailored to the needs of aging populations in intelligent wellness contexts. How to leverage technological innovation to promote service integration and enhance full-cycle delivery efficiency remains an urgent and unresolved issue.

In response, this study investigates both the design logic and empirical effectiveness of an SMT-integrated intelligent wellness system, developed within the broader framework of digital transformation and population aging. A comprehensive analysis was conducted, encompassing the system’s theoretical foundation, user demand architecture, functional modules, and operational mechanisms. Field validation was performed via empirical research involving older adult populations in Jinan, Qingdao, and Rizhao—three cities in China. This research ultimately seeks to propose a replicable and sustainable system design framework for SMT-integrated wellness services. It offers both theoretical insight and actionable pathways to support the evolution of China’s sports-centered health industry and the implementation of its active aging strategy.

## 2 Literature review

In the context of an aging society and the rise of digital technologies, the integration of SMT into an older adult Care and Health System is examined as a new paradigm for older adult services. Within the intertwined relationship between older adult care as a public service and an industry, this section analyzes the foundations, motivations, and application trends of the intelligent older adult care and health concept, while clarifying the boundaries between government and the market. The goal is to integrate intelligent technologies and system design concepts into the older adult care industry, linking older adult care products and services to facilitate the continuous flow and sharing of data, alleviate human resource pressures, and shift societal perspectives on older adult care.

### 2.1 Transition of traditional sport and older adult care service models

China’s traditional sport and older adult care service model has undergone a long process of development. It started with the emergence of basic concepts, progressed to the formation of service content, and eventually evolved into a system. The concept of integrating sports, medicine, and older adult care services that we know today emerged from the combined influence of social, technological, and cultural factors. Historically, the gradual development of the sport and older adult care service model has been shaped by three key factors: social context, policy direction, and technological integration. However, as the aging process accelerates, the phenomenon of “growing old before getting rich” has become more apparent in China. Outdated older adult care concepts have led to weak awareness among the older adult regarding scientific older adult care and health management. The influence of traditional thinking and ingrained habits has made the promotion of intelligent older adult care and health systems challenging (11).

**Figure 1:**
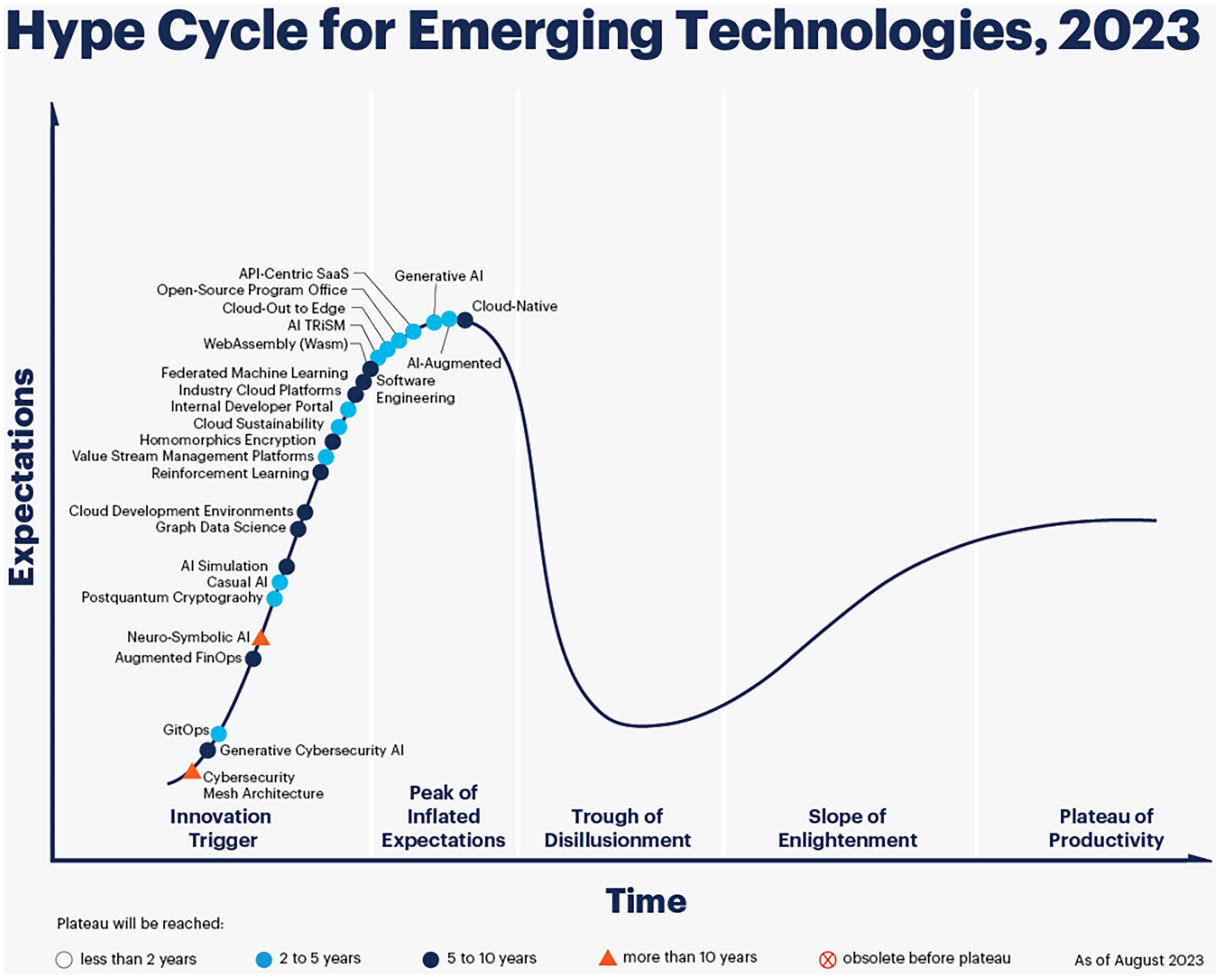
Gartner’s 2023 emerging technology maturity curve. Source: Gartner Consulting Company.

Fortunately, despite the growing trend of aging, we have also entered the digital era. Gartner’s 2023 emerging technology maturity curve (Figure 1), 25 representative technologies that are transitioning from physical to intelligent were identified (12). Currently, intelligent technology plays a vital role in the field of sport and older adult care services, making technology omnipresent, constantly available, and interconnected with business ecosystems.

The advancement and application of internet technologies have accelerated the deep integration of the “SMT” sport and older adult care service industry with the broader service sector, stimulating the emergence and innovation of new older adult care service models (13). Based on this, this paper categorizes sport and older adult care service models into three types: traditional sport and older adult care service models, smart sport and older adult care service models, and intelligent sport and older adult care service models (Table 1).

**Table 1:** Sport and older adult care service models in China.

Model	Traditional sport and older adult care service models	Smart sport and older adult care service models	Intelligent sport and older adult care service models
Background	Early stage of population aging, initial formation of older adult care service system, limited application of technology	Rapid technological development, older adult care services begin to incorporate smart technology	High development of digital and intelligent technologies, comprehensive upgrade of older adult care services
Venue	Fixed locations such as parks, gyms, stadiums	Various settings like homes, communities, and professional institutions	Virtual spaces created by VR, AR combined with physical facilities
Concept	Centered on traditional Chinese medicine wellness concepts, focusing on physical exercise and lifestyle adjustments	Emphasis on technology application, focusing on personalized services and data monitoring	Centered on holistic human needs, emphasizing intelligent services and humanistic care
Perspective	Passive acceptance, reliant on family and community support	Shift from passive acceptance to active participation, attention to personal health data	Equality and respect, focusing on self-fulfillment and social value of the older adult
Needs	Basic physiological needs such as diet, living, and safety	Primarily physical health needs, with growing attention to mental health and social needs	Highly customized, dynamically adjusted services to meet changing user needs
Content	Traditional sports programs like Tai Chi, Baduanjin, dietary adjustments, traditional Chinese massage	Smart fitness equipment monitors health data, offers personalized exercise plans, remote medical services	Personalized exercise plans based on big data analysis, intelligent rehabilitation training, older adult care and health experiences integrating culture and tourism
Actors	Primarily led by coaches or instructors	Users participate in health management themselves	Collaboration among multiple roles including AI assistants, remote doctors, in addition to the user
Technology	Traditional sports techniques, like Tai Chi moves and Chinese massage methods	Smart sports sensors, remote medical technology, etc.	Advanced technologies such as AI, IoT, big data, forming an interconnected technological ecosystem
Resources	Mainly human resources including medical staff, rehabilitation therapists, and traditional wellness foods and herbs	Smart devices, big data platforms, etc.	Comprehensive resource integration, leveraging cloud platforms to share high-quality global healthcare, tourism, cultural, and health resources

Source: compiled by the author.

### 2.2 The basic concept of “SMT” intelligent integration in older adult care and health

The “SMT” intelligent integration system for older adult care and health represents a systematic manifestation of the intelligent older adult care concept in the context of an aging society and the digital age. Driven by industry and real needs of the older adult, this system establishes connections between products and services, promotes the flow and sharing of data resources, and strives to create an intelligent and sustainable older adult care and health service. This system skillfully integrates resources from multiple fields, including sports, medicine, and tourism, to build a data-driven service-sharing platform that strongly promotes the transformation and upgrading of the older adult care service industry.

Currently, researchers have different understandings of intelligent older adult care and health, but they all recognize that it has both social and technological attributes (14, 15). In practice, intelligent older adult care and health has moved beyond the traditional government-led model, gradually evolving into a collaborative system led by businesses, guided by the government, and actively participated in by social organizations. The service goals have expanded from merely providing basic living security to also focusing on value realization, industry development, and technological optimization, in active response to the national strategy for positive aging.

From a design perspective, the “SMT” intelligent integration system for older adult care and health aims at industry upgrading, focusing on creating a resource-sharing platform driven by user needs. On the one hand, physical are used to collect behavior data from older adult users, identify potential needs, and facilitate the flow and sharing of resources. For example, smart devices with motion monitoring functions can be developed to track older adult people’s physical activity, providing them with personalized exercise recommendations and healthcare plans (16). On the other hand, enterprises tad in resource allocation, the government is responsible for oversight and coordination, and social organizations act as connecting channels, working together to drive system development. This system emphasizes both “caring for the older adult” and “respecting the older adult,” aiming to meet the health and wellness needs of the older adult while tapping into their wisdom to enable them to contribute meaningfully and live peacefully (17).

To address current issues such as the isolated fragmentation of intelligent older adult care and health products and high service costs, the “SMT” system employs strategies like identifying core users, clarifying government responsibilities, and establishing connections between products and services. These efforts ensure product iteration and precise service matching, creating a positive cycle of development.

### 2.3 The theoretical foundations of “SMT” intelligent integration in older adult care and health

**Figure 2:**
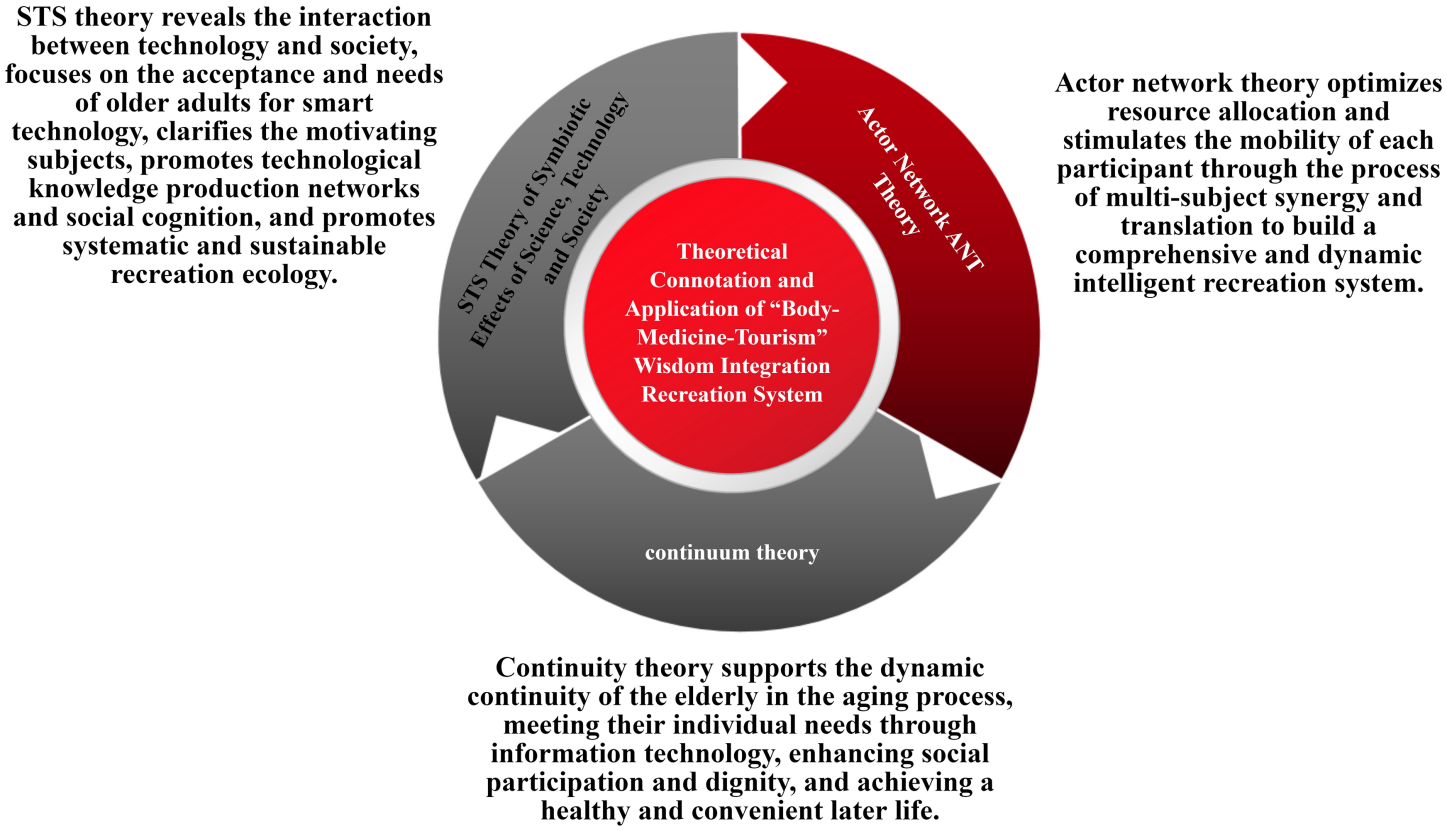
The theoretical connotations and applications of the “SMT” intelligent integration system for older adult care and health. Source: this is drawn from the author’s collated materials.

The design of the “SMT” intelligent integration system for older adult care and health should move beyond traditional frameworks, integrating content from multiple disciplines and shifting from macro-level to micro-level thinking. In the digital information era, it is essential to explore the relationship between technological integration, social harmony, and user utility. This study is grounded in the STS (Science, Technology, and Society) theory of symbiosis between technology and society, rethinking the relationship between technology and society while reconstructing the system elements (18). Actor-Network Theory is applied to establish system hierarchies and coordinate actors, while Continuity Theory is employed to build a dynamic relationship between older adult users and society (Figure 2).

## 3 Methodology

Existing information management platforms often overlook the true understanding and analysis of older adult individuals’ needs, as well as their ability to use intelligent technologies and products. Therefore, the “SMT” intelligent integration system for older adult care and health focuses on the relationships between stakeholders and key drivers within the system, with an emphasis on the overall architecture. Targeting “younger older adult” as the core breakthrough user group, the system design principles are analyzed from the perspective of micro-level need recognition and need hierarchy. This section conducts a detailed analysis of the micro-level needs, investigates the components of system design based on the service gaps in current platforms, and examines the internal drivers, functions, and mechanisms of the system’s design. The aim is to explore ways to enhance the system’s ability to meet demand, improve older adult users’ awareness, usage, and acceptance of the system (19, 20).

### 3.1 Understanding and analyzing the needs of “SMT” intelligent integration for older adult care and health

Macro-level planning and management primarily focus on addressing the needs of older adult populations through governmental, community, and institutional approaches, often failing to delve into the micro-level needs of individuals. As a result, older adults find it difficult to directly benefit from these services. The design of the “SMT” intelligent integration system for older adult care and health is based on a deep understanding of older adult care needs, analyzing the identification and categorization of micro-level needs in the Chinese context, and providing a solid foundation for studying the system’s mechanisms (Table 2).

**Table 2:** Perceptions of micro-needs hierarchy of “sport-medicine-tourism” intelligent integrated recreation system.

Type of requirement	Specification	Typical example
Physical health needs	Daily physical exercise, disease prevention	The system uses smart sports bracelets to monitor exercise data and provide personalized exercise advice; it monitors health through smart medical devices and organizes rehabilitation activities such as tai chi and goalball
Mental health needs	Social activities, counseling services	Organize senior sports clubs, fun sports competitions; provide professional psychological counseling and encourage family and volunteer support
Demand for social participation	Community activities, volunteerism	Organize senior square dance competitions, fitness walks; provide sports volunteer opportunities
Quality of life needs	Comfortable living environment and convenient transportation	Provide age-appropriate living environment and sports facilities; provide convenient transportation to sports venues and tourist attractions
Ownership needs	Self-care ability in daily life, ability to use smart devices	Provision of daily living aids; training in the use of sports-smart devices

#### 3.1.1 Accurate understanding of micro-level needs

Traditional older adult care services often focus on macro-level management and planning, overlooking individual differences and specific needs (21). Intelligent integration older adult care and health system approaches this from a micro perspective, integrating sports, medicine, and tourism to provide personalized and precise services for the older adult. These needs span multiple dimensions:

(1) Physical health needs: including daily exercise and disease prevention. For instance, the system can use intelligent fitness trackers or wearable devices to monitor older adult individuals’ activity data and provide personalized exercise recommendations based on their physical condition. It can also monitor health through smart medical devices, detect potential health issues, and organize sports rehabilitation activities suited for the older adult, such as Tai Chi and gateball, to enhance physical fitness.

(2) Psychological health needs: encompassing social activities and psychological counseling services. The system can organize various sports-related social activities, such as senior sports clubs and fun athletic competitions, allowing older adult individuals to make friends and alleviate loneliness through exercise (22). It can also offer professional psychological counseling services and encourage emotional support from family members and social volunteers (23).

(3) Social participation needs: involving community activities and volunteer services. The system can organize diverse community sports activities and interest groups, such as senior square dancing competitions and fitness walking activities, encouraging older adult people to participate actively. It can also provide volunteer opportunities in sports, allowing older adult individuals to serve as instructors for community sports events.

(4) Quality of life needs: including comfortable living environments and convenient transportation. The system can provide senior-friendly housing combined with sports facilities, such as community fitness trails and senior parks, enabling easy access to daily exercise. It can also offer convenient transportation services, making it easier for seniors to visit sports facilities and tourist destinations.

(5) Autonomy needs: covering daily self-care abilities and the use of smart devices. The system can provide daily living aids and offer training on using smart sports devices, such as exercise apps that guide seniors in their workouts, thus enhancing their independence and autonomy.

#### 3.1.2 Detailed micro-level needs analysis

A thorough understanding of older adult people’s micro-level needs allows for more precise analysis and better system design and implementation. For physical health, in addition to monitoring and exercise recommendations, the system can combine medical resources to provide rehabilitation services tailored to different health conditions. Regarding psychological health, sports social activities not only help alleviate loneliness but also boost confidence and a sense of accomplishment. Psychological counseling services can be combined with physical activity to relieve stress. For social participation, sports can be an essential way for the older adult to integrate into society. Community activities and interest groups can spark vitality and creativity, while volunteer services help them feel valued. For quality of life, combining senior-friendly living environments with sports facilities provides a comfortable living space and easy access to exercise. Convenient transportation allows the older adult to participate in sports and tourism activities. For autonomy, training on smart sports devices improves their acceptance and use of new technology, enhancing their confidence in independent living.

#### 3.1.3 Responding to micro-level expectations

When designing the “SMT” intelligent integration system for older adult care and health, it is crucial to consider older adult people’s expectations for their future lives. Seniors hope that smart technology will enhance their quality of life by offering personalized sports, medical, and tourism services, providing easy-to-use experiences, and ensuring safety and reliability. Additionally, they seek social recognition and respect, wishing to gain social approval through participation in sports volunteering and social activities. They also expect the system to continue evolving and improving by introducing new technologies and services to meet their changing needs. By comprehensively understanding and analyzing these micro-level needs and expectations, the “SMT” intelligent integration system can better provide intelligent, personalized, convenient, safe, and socially recognized services, ultimately helping older adult individuals achieve a healthy, happy, and dignified later life.

### 3.2 The mechanism and elements of the “SMT” intelligent integration for older adult care and health

The “SMT” intelligent integration system for older adult care and health is a comprehensive service system that integrates sports, medicine, and tourism to provide older adult individuals with personalized and holistic care through the application of intelligent technology. It emphasizes the integration of sports elements, the deep connection between sports and medicine as well as sports and tourism, and the seamless fusion of all three to achieve centralized feedback and information processing. The system is driven by the real needs of the older adult, addressing both universal needs (such as physiological and cognitive compensation) and personalized needs, which are further divided into active, unactivated, and potential needs. Through big data analysis, it identifies hidden communities among older adult users, enabling precise service delivery.

In addressing the demand for older adult care, the integration of sports elements is emphasized. Given the physical characteristics of older adults, the system incorporates a range of tailored physical activities, such as rehabilitative exercises and senior fitness programs. These activities enhance physical health, improve coordination and balance, prevent illness, and promote social interaction, ultimately improving both quality of life and autonomy (24). Physical products, as front-end components, are designed to meet the behavioral preferences of older adult users through age-appropriate features, reducing the learning curve and enhancing user engagement. For instance, smart wearable devices with sports functionalities not only monitor health data but also track physical activities, such as steps and intensity, providing personalized exercise recommendations.

The ultimate goal of the system is to create an intelligent environment by leveraging IoT and cloud computing technologies to break down the barriers of traditional elder care models, establishing a comprehensive, multi-dimensional active aging environment. The system’s mechanism consists of three core elements: “mind–body synergy,” “layered strategies,” and “symbiotic links.”

(1) Mind–body synergy: The system dynamically adapts to changes in physical, medical, and travel preferences by providing personalized healthcare plans and suitable travel destinations based on the user’s health and activity preferences.

(2) Layered strategies: Through age-appropriate product design, it addresses varying levels of needs. Products are developed with both low-tech accessibility and high-tech flexibility in mind, such as creating smart rehabilitation fitness equipment that combines exercise and medical rehabilitation functions, helping seniors bridge the digital divide.

(3) Symbiotic links: By integrating sports, healthcare, and tourism industries, the system fosters a sustainable ecosystem. Collaborative efforts between these sectors promote the expansion of the wellness industry and contribute to societal development. For example, sports training institutions partner with senior living communities to offer fitness programs, healthcare providers ensure medical support during activities, and tourism companies develop travel packages tailored to older adult participants.

**Figure 3:**
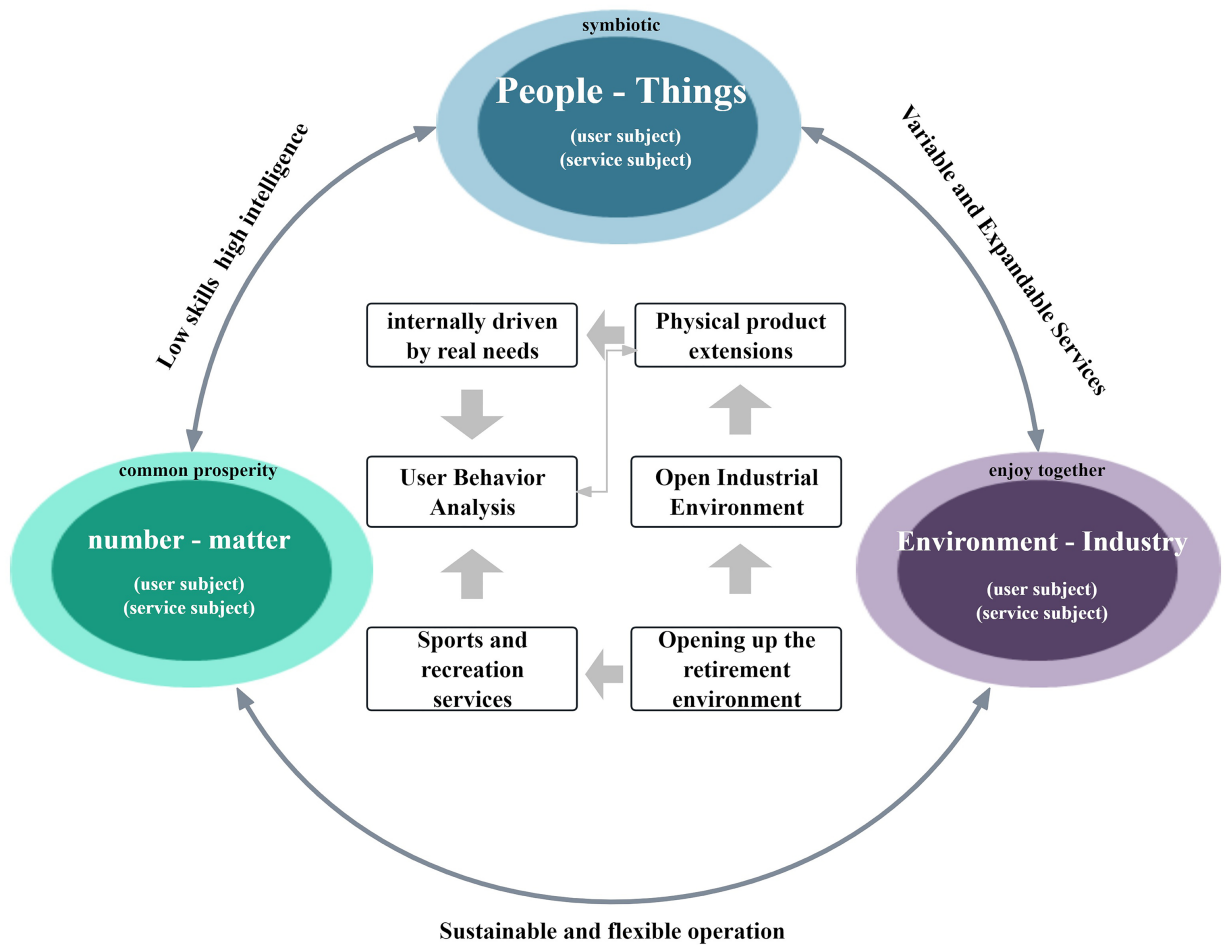
Design elements of the intelligent integrated health system for “SMT”. Source: this is drawn from the author’s collated materials.

The “SMT” Intelligent Integrated older adult care and health system aims to create a dynamic, collaborative, and multi-level coordinated active aging service framework (Figure 3). Utilizing digital information technologies, it links physical products with virtual services to comprehensively meet the multi-layered needs of the older adult population. Compared to traditional elder care models, this system shifts from government-led to industry-driven, diversifies service recipients, and extends service content from primarily caregiving to a broader range of needs, enabling two-way information exchange.

Based on the Science, Technology, and Society (STS) Symbiosis Theory, the system’s key drivers include older adult individuals and their families, enterprises, governments, social organizations, virtual services, and physical products. Through data extraction and analysis, these entities foster effective internal communication and collaboration, enhancing the system’s sustainability (25). For instance, businesses can develop innovative products and services based on data collected on the older adult’s physical activity needs, while governments ensure smooth system operation through policy guidance and resource allocation.

The system’s design comprises three core symbiotic layers: human-product symbiosis, event-data symbiosis, and environment -industry symbiosis:

(1) Human-product symbiosis layer: this focuses on matching the needs of the target population with physical products. The goal is to ensure that products fulfill the practical requirements of older adult individuals in physical activity, healthcare, and travel. For example, specialized sports equipment, medical devices, and travel accessories like lightweight wheelchairs, portable oxygen tanks, and travel canes are developed to optimize their functionality for seniors (26).

(2) Event-data symbiosis layer: this layer ensures rapid response and precise services through event management and data analysis. For instance, if an older adult individual experiences a health issue during physical activity, the system can quickly retrieve the data and arrange medical assistance. Similarly, during travel, the system adjusts itineraries and services based on user feedback, ensuring both safety and enjoyment in sports-related experiences.

(3) Environment-industry symbiosis layer: this layer promotes cross-industry integration to address the diverse needs of home-based older adult care environments. Recognizing differences in living environments, the system integrates sports, medical, and tourism resources to provide tailored services. For example, in community elder care settings, the system organizes community sports activities, sets up community medical service stations, and offers local cultural tourism programs, allowing seniors to enjoy the benefits of sports, healthcare, and tourism within familiar surroundings.

## 4 Framework and methodological development of the intelligent integration of “SMT” in the older adult care and health system design

In the previous two chapters, this study has provided a detailed discussion on the fundamental framework and theoretical foundations of the intelligent integration of SMT in the older adult care and health system design. It has also conducted an in-depth analysis of the system’s micro-level demand recognition, analytical foundations, structural mechanisms, and key components, laying a solid theoretical and empirical foundation for the framework and methodological development presented in this chapter. Building upon these insights, this chapter systematically constructs the framework of the SMT-integrated older adult care and health system by establishing system objectives, developing design methodologies, and analyzing system architecture. Furthermore, it formulates a comprehensive system model and provides an in-depth discussion of its operational mechanisms.

### 4.1 Architectural framework of the intelligent integration of “SMT” in the older adult care and health system design

#### 4.1.1 Establishment of system objectives for the intelligent integration of “SMT” in the older adult care and health system design

The goal system of the Intelligent Integration of SMT in the older adult Care and Health System Design is divided into three progressive categories: system mechanism objectives, design philosophy objectives, and social value objectives.

##### 4.1.1.1 Deep integration: system mechanism objectives

The primary objective is to establish a deep integration mechanism for SMT, breaking down industry barriers and enabling collaborative development. By integrating multiple elements and functions, the system creates a synergistic supply effect to meet the diverse needs of older adult users. In traditional older adult care models, the micro-level needs of older adult users are often overlooked. However, this system addresses this issue by recording and analyzing user behavior data to accurately match demand with supply, thereby enhancing service quality. The deep integration mechanism requires collaborative interaction among all stakeholders (suppliers, users, regulatory bodies, etc.), driving the transformation of older adult care services from a fragmented to a holistic, interactive model and fostering industrial innovation.

##### 4.1.1.2 Health first: design philosophy objectives

“Health First” is the core design philosophy of the system, aimed at enhancing the quality of life for the older adult population. The design focuses on the comprehensive life scenarios of older adult individuals, utilizing smart technologies to reduce learning costs and ensure the autonomy of older adult people in terms of their physical, psychological, and social capabilities. The system design addresses not only the physiological needs of the older adult but also their psychological needs, social interactions, and self-fulfillment. Through a tiered, graded response mechanism, it meets diverse requirements. Additionally, the system emphasizes the strengths and capabilities of older adult individuals, conveying their life knowledge and social experience through design to enhance their sense of dignity.

##### 4.1.1.3 Industry collaboration: social value objectives

The ultimate goal of the system is to enhance the social collaboration capacity of the older adult care industry and promote the harmonious development of an aging society. Through digital means, the system breaks away from the traditional single-service model in the older adult care industry, fostering service diversification and resource sharing. The older adult are not only the recipients of care but also active contributors to the system’s construction, reflecting the principles of equality and respect. Through collaborative efforts, various supply entities within the system create a pattern of resource sharing and value co-creation, driving the sustainable development of the older adult care industry, expanding the silver economy, and ultimately achieving the harmonious development of an aging society.

#### 4.1.2 Methodological development of the intelligent integration of SMT in the older adult care and health system design

The construction of the design methodology for the Intelligent Integration of SMT in the older adult Care and Health System spans the entire process, including the organization of collaborative elements, system functional layering, system operation design, and supply development.

##### 4.1.2.1 Design guidance based on age-friendly principles

The system design must address the diverse physiological, psychological, and behavioral needs of the older adult, adhering to age-friendly principles such as safety, usability, comfort, and accessibility. By utilizing smart technologies for data processing, the system enhances the “natural interaction” aspect of products and services, with a particular emphasis on age-friendly cognitive design. The system should be based on the psychological and behavioral characteristics of the older adult, projecting their behavioral patterns and sensory traits into product designs. It should offer various forms of interaction, such as physical interfaces, graphical interfaces, and voice interfaces, to provide tailored services to older adult users.

##### 4.1.2.2 System design methodology and strategies

The construction of the system architecture should be approached from the perspective of technology and societal symbiosis, establishing the relational structure of the macro external environment of the system. System design is a result of the combined influence of societal, economic, and technological factors, including trends in aging societies, transformations in the older adult care industry, and advancements in smart technologies. By analyzing these macro factors, the design aims at industrial upgrading, establishing effective and dynamic collaborative relationships among elements, thereby enhancing the system’s industrial inclusivity and sustainability. The study of system mechanisms involves extensive interaction analysis among system elements, which are complex and diverse, encompassing subjective and objective factors, human and non-human elements, and their interdependencies within the collaborative mechanism. This results in a multi-centered service ecosystem.

#### 4.1.3 The architectural structure of the intelligent integration of SMT in the older adult care and health system design

**Figure 4:**
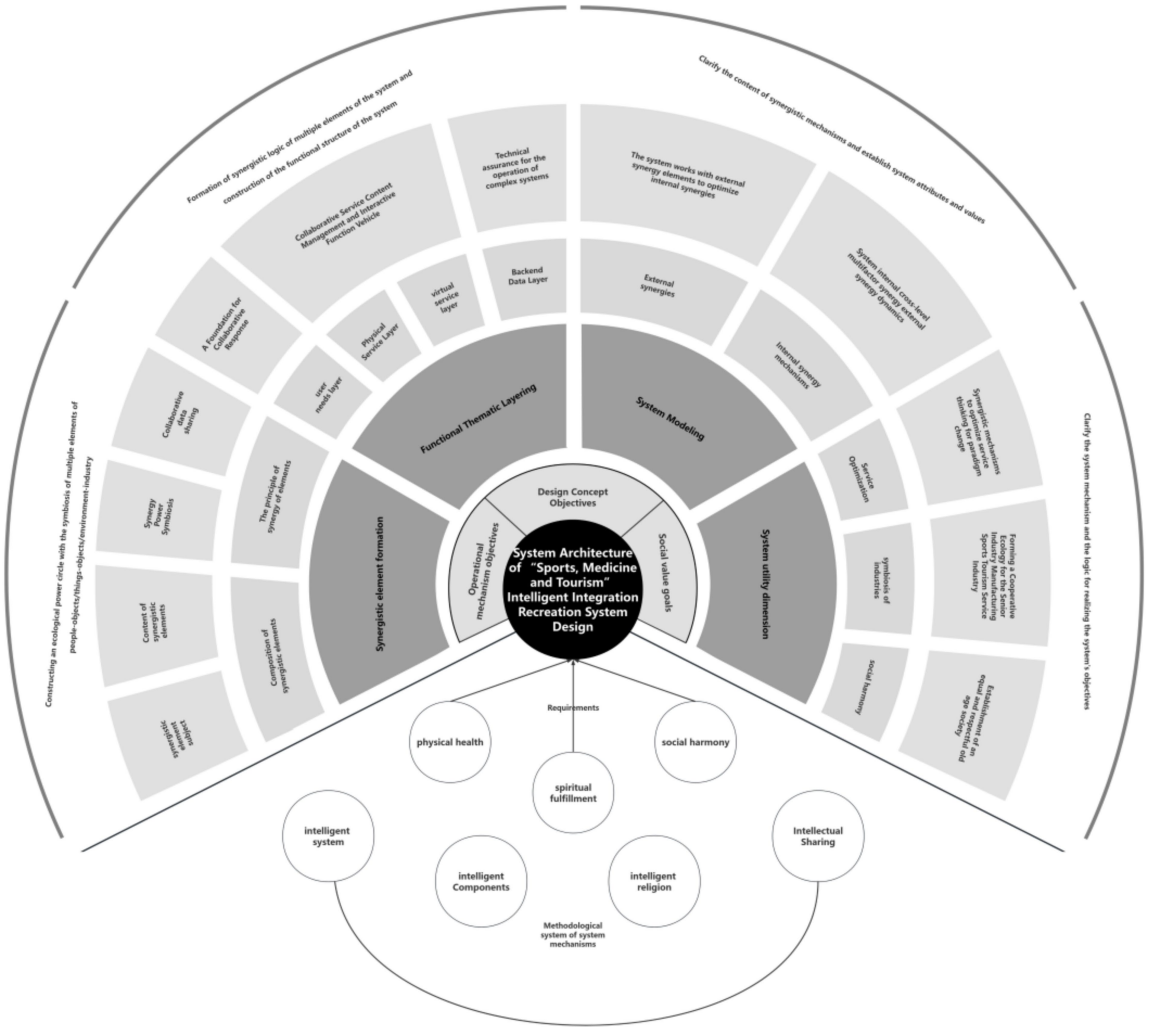
System architecture of “Sport, Medicine and Tourism” intelligent integration recreation system design. Source: this is drawn from the author’s collated materials.

The architectural structure of the Intelligent Integration of SMT in the older adult Care and Health System is composed of four key components: collaborative elements, functional theme layering, system utility dimensions, and system collaboration mechanisms. These four elements correspond to the system’s content, functions, values, and operational model, respectively (Figure 4).

##### 4.1.3.1 Formation of collaborative elements

Collaborative elements consist of the main stakeholders and the content of collaboration. The main stakeholders include the older adult population, corporate partners, service providers, government, and social organizations. The content of collaborative elements revolves around these stakeholders, with the older adult as the core recipients of services, and their feedback constituting the central content. Corporate partners, as suppliers, align their offerings with the system’s demands. Service providers, as the carriers of system functions, refine the system’s service content. The government and social organizations act as guarantors, ensuring the regulation and assurance of the system’s older adult care services.

##### 4.1.3.2 Functional theme layering

The system is divided into three layers based on different functional entities: the user demand layer, the backend data layer, and the perception application layer. The user demand layer defines the system’s target audience and the necessary service content. The perception application layer includes both software applications providing virtual services and physical service products. It integrates hardware and software to support service management and delivery. The backend data layer ensures the operation of the system’s “smart” shared collaboration mechanism from a technical perspective, facilitating data collection and analysis to support the flow and sharing of data among internal and external collaborative entities.

##### 4.1.3.3 System model construction

The system design methodology constructs a system mechanism framework through the overall analysis of system elements, their collaborative processes, and content. This framework consists of intelligent systems, smart components, intelligent environments, and intelligent sharing. The user demand layer serves as the prerequisite for the intelligent environment’s service needs, while the backend data layer provides technical support for the intelligent environment’s operation. The operational process of the intelligent environment delivers the content for intelligent sharing, and external collaborative entities within the system mechanism serve as the sharing subjects, collectively establishing the value transmission mechanism of the system.

##### 4.1.3.4 System utility dimensions

The system design framework, based on the system objectives, integrates system design strategies, and builds a collaborative model around a multi-element, multi-level structure. It has significant implications in three key areas: innovation in older adult care models, enhancement of the social value of the older adult care industry, and the promotion of respect for the older adult. By constructing internal and external collaboration mechanisms, the system ensures that government, enterprises, social organizations, and other older adult care service providers can convey and apply their value in service delivery, thus facilitating the creation of a collaborative ecosystem between manufacturing and service industries within the system.

### 4.2 Driving mechanisms of the intelligent integration of “SMT” in the older adult care and health system design

#### 4.2.1 Integrated innovation: a health promotion system for the entire life cycle

Within the framework of life cycle management, the “SMT” intelligent integrated older adult care and health system offers a novel approach to comprehensive health management, spanning prevention, treatment, and rehabilitation. This system not only addresses physical health but also extends to psychological well-being and social adaptability, forming a holistic health promotion model. Through physical activities such as regular senior fitness exercises and Tai Chi classes, the system enhances the physical functions of the older adult while also fostering social interactions and bolstering their psychological resilience. Medical services play a pivotal role in this system, providing not only routine health check-ups but also telemedicine consultations and emergency rescue services, ensuring that the older adult receive timely and effective care when faced with health issues.

The “SMT” older adult care and health system cleverly incorporates tourism into health management by designing several scenic routes tailored to seniors, integrating health elements such as hiking, cycling, and wellness seminars. This allows the older adult to enjoy the pleasures of travel while simultaneously nurturing both body and mind. Additionally, the system promotes health awareness through regular health lectures and knowledge dissemination, helping seniors adopt healthier lifestyles. This integrated “SMT” older adult care and health model is also introduced into urban communities. Communities are equipped with professional sports facilities and coaches who provide personalized fitness programs for seniors. Additionally, on-site medical stations offer daily healthcare services and emergency assistance, while regular tourism activities encourage seniors to explore nature, enriching their lives. Through this model, the quality of life for the older adult has significantly improved, with better physical health and enhanced psychological well-being.

In summary, the “SMT” intelligent integrated older adult care and health system, through its life cycle management, organically combines sports, medicine, and tourism to provide comprehensive and multi-dimensional health promotion services. It lays a solid foundation for enhancing the quality of life for the older adult and promoting healthy aging.

#### 4.2.2 Intelligent response: a smart support system for personalized services

In the “SMT” intelligent integrated older adult care and health model, the application of big data and artificial intelligence (AI) technologies provides robust support for delivering personalized services. By collecting and analyzing data on seniors’ health, physical activity, and tourism preferences, the system employs advanced algorithmic models to tailor individualized health management plans, exercise programs, and travel recommendations for each user. This personalized approach not only enhances the precision of services but also significantly improves user satisfaction and engagement. Smart devices and applications play a critical role in the system. Wearable devices, such as heart rate monitors and blood pressure sensors, can track seniors’ physiological indicators in real-time and transmit the data to the system’s backend. By analyzing this data, the system can identify potential health risks and provide timely health alerts to users. Additionally, mobile applications act as a bridge between users and the system, offering a convenient interface through which seniors can access their health data, schedule medical appointments, participate in physical activities, and plan travel itineraries.

The integration of big data and AI technologies, along with the widespread use of smart devices and mobile applications, presents new development opportunities for the “SMT” intelligent integrated health system. The intelligent response mechanism plays a crucial role in enhancing service flexibility and precision. Based on seniors’ health status, exercise habits, and travel preferences, the system can dynamically adjust its service offerings to ensure personalization and effectiveness. Moreover, by incorporating user feedback, the system continuously optimizes service processes and quality, improving the overall user experience and meeting the evolving needs of seniors. This drives innovation in the older adult care and health industry.

#### 4.2.3 Collaborative development: building an ecosystem with multi-stakeholder participation

In the “SMT” intelligent integrated older adult care and health model, the collaborative efforts of various stakeholders—government, enterprises, social organizations, and senior users—are key drivers of the older adult care and health industry’s growth. The government provides strong support by establishing relevant policies and offering financial assistance to ensure the industry’s development. Enterprises leverage their technological expertise and market experience to provide advanced technological support and a wide array of services to the system. Social organizations contribute by organizing public welfare activities and offering volunteer services, thus extending additional care and support to seniors. As direct beneficiaries of the system, seniors play a vital role, with their needs and feedback serving as critical drivers for the system’s continuous optimization and improvement.

An open platform and resource sharing are essential mechanisms to foster multi-stakeholder collaboration. Through the creation of an open platform, the government, enterprises, and social organizations can easily share information, exchange experiences, and collaborate on technological advancements, thus forming an ecosystem of collaborative innovation. This spirit of openness and sharing not only strengthens cooperation among stakeholders but also accelerates the growth of the health and older adult care industry.

In terms of sustainable development, the “SMT” intelligent integrated older adult care and health model achieves a win-win outcome in both economic and social benefits by optimizing resource allocation and enhancing service efficiency. Through precise service matching and efficient resource utilization, the system reduces service costs and improves service quality. Additionally, the system fosters health awareness and healthy lifestyles among seniors, promoting overall societal well-being. This sustainable development model not only provides new opportunities for the older adult care and health industry but also makes a significant contribution to building a harmonious society.

## 5 Experimental and measurement evaluation of the intelligent integration of SMT in the older adult care and health system design

### 5.1 Experimental design

#### 5.1.1 Participants

This study collected experimental samples from three cities in China—Jinan, Qingdao, and Rizhao—considering regional differences in economic development, cultural environment, and older adult care resource distribution. Jinan is characterized by abundant medical resources and a rich cultural heritage; Qingdao boasts a developed economy and a thriving tourism industry; Rizhao offers an excellent ecological environment with high livability. The study was conducted in collaboration with local communities and civil affairs departments, employing a stratified random sampling method. Participants were stratified based on age (60–70 years, 71–80 years), health status (generally healthy, mild chronic disease, moderate chronic disease, severe chronic disease), and economic income level (high, middle, low). A total of 300 older adult individuals were randomly selected from these stratified groups and assigned to either the experimental group or the control group, with 150 participants in each. After grouping, statistical tests were conducted on variables such as age, health status, and economic income to ensure no significant differences between the two groups (*p* > 0.05), thereby guaranteeing the comparability and reliability of the experiment.

The experimental period was set at 12 months, comprising three phases: pre-test, intervention, and post-test. The pre-test phase was conducted within 1 week before the experiment to measure baseline indicators for both groups. The intervention phase lasted 10 months, during which the experimental group received services from the Intelligent Integration of ‘SMT’ in the older adult Care and Health System, while the control group received traditional older adult care services. The post-test phase was conducted within 1 week after the intervention period, during which all indicators were reassessed. The data from both groups were compared and analyzed to evaluate the intervention effects of the Intelligent Integration of ‘SMT’ in the older adult Care and Health System.

#### 5.1.2 Experimental tools

##### 5.1.2.1 Physical health indicators

Professional medical testing equipment was used to measure the participants’ physical health indicators, including:

- Blood pressure: Measured using an electronic sphygmomanometer to assess systolic and diastolic blood pressure.
- Blood glucose: Fasting blood glucose and postprandial two-hour blood glucose levels were measured using a glucometer.
- Blood lipids: Total cholesterol, triglycerides, low-density lipoprotein cholesterol, and high-density lipoprotein cholesterol were assessed using a biochemical analyzer.
- Cardiopulmonary function: Measured using a cardiopulmonary function tester, including indicators such as vital capacity and maximal oxygen uptake.
- Physical activity ability: Evaluated using the Activities of Daily Living (ADL) scale, assessing the participants’ ability to perform daily activities such as dressing, eating, bathing, and walking (Table 3).

**Table 3:** Measurement methods and standards for physical health indicators.

Norm	Gauge	Measurement methods	Standard of measurement
Systolic blood pressure, diastolic blood pressure	Sphygmomanometer	Let the older adult rest quietly for 5–10 min, take the sitting position, measure the blood pressure of the right upper arm, measure 3 times consecutively and take the average value.	Normal range: systolic blood pressure 90–139 mmHg, diastolic blood pressure 60–89 mmHg
Fasting blood glucose, 2-h postprandial blood glucose	Glucose meter	Measurement of fasting blood glucose after 8–12 h of fasting; 2-h postprandial blood glucose measurement after 2 h of eating the first bite of food	Normal range of fasting blood glucose: 3.9–6.1 mmol/L, normal range of postprandial 2-h blood glucose: <7.8 mmol/L
Total cholesterol, triglycerides, LDL cholesterol, HDL cholesterol	Biochemical analyzer	Venous blood is drawn for testing	Normal range of total cholesterol: <5.2 mmol/L; normal range of triglycerides: <1.7 mmol/L; normal range of LDL cholesterol: <3.4 mmol/L; normal range of HDL cholesterol: >1.0 mmol/L for men, >1.3 mmol/L for women
Lung capacity, maximal oxygen uptake	Cardiopulmonary function tester	Follow the device’s operating instructions and have the older adult perform the appropriate breathing and exercise tests	There are different normal reference ranges depending on age and gender
Physical activity	Activities of Daily Living Scale (ADL)	Scoring by asking and observing older adult people’s self-care in 10 areas such as dressing, eating, bathing and walking	A total of 100 points, with higher scores indicating better self-care skills

##### 5.1.2.2 Psychological health indicators

In this study, psychological health was assessed using two scales: the Geriatric Depression Scale (GDS-15) and the Satisfaction with Life Scale (SWLS), providing a comprehensive evaluation of older adult participants’ mental well-being. The GDS-15 consists of 15 items with “yes” or “no” responses, yielding a score range of 0–15, where higher scores indicate greater levels of depression (27). Reliability analysis in this study showed that the Cronbach’s *α* coefficient was 0.82 in the pre-test and 0.85 in the post-test, demonstrating high internal consistency and the scale’s reliability in assessing depressive symptoms among older adults. The SWLS consists of five items rated on a 7-point scale, with higher scores reflecting greater life satisfaction (28). Reliability analysis in this study indicated a Cronbach’s α coefficient of 0.88 in the pre-test and 0.90 in the post-test, confirming strong internal consistency and the scale’s suitability for evaluating life satisfaction among older adults.

##### 5.1.2.3 Social participation indicators

Older adult individuals’ social participation was assessed through a questionnaire survey focusing on three key aspects:

- Participation in community activities. Data were collected on involvement in various community events, such as cultural performances and volunteer services. Community staff recorded participation details (time, content, and attendees) on a monthly basis, with confirmation from both organizers and participants to ensure data accuracy.
- Membership in interest groups. The number of groups older adult individuals joined was recorded through a questionnaire to gauge their engagement in different interest areas.
- Size of social networks. The number of close friends and relatives with whom participants maintained regular contact was used as a measure of social connectedness. To ensure data reliability, pre-test and post-test questionnaires were carefully designed to minimize leading or ambiguous questions.

Through these dimensions, the study comprehensively assessed older adult individuals’ social participation, providing robust data support for the research.

##### 5.1.2.4 Quality of life indicators

The study employed the World Health Organization Quality of Life-BREF (WHOQOL-BREF) scale to comprehensively evaluate the quality of life of older adult individuals (Table 4). The scale consists of 26 items covering four dimensions: physical health, psychological well-being, social relationships, and environment, rated on a 5-point scale, with higher scores indicating better quality of life. During the pre-test and post-test phases, trained investigators guided participants in completing the questionnaire. For those with reading or comprehension difficulties, responses were collected through verbal interviews. The WHOQOL-BREF scale has undergone translation and cultural adaptation for use in the Chinese older adult population. Scoring was conducted by assigning a value between 1 and 5 to each item based on participants’ responses. The score for each dimension was calculated as the average score of all items in that dimension multiplied by four, while the total score was the sum of all item scores. Reliability analysis showed that Cronbach’s *α* coefficients for all dimensions exceeded 0.7, indicating good reliability and providing a scientific basis for improving older adult individuals’ quality of life.

**Table 4:** Distribution of WHOQOL-BREF scale dimensions and items.

Dimension	Number of projects	Thrust
Physiological field	7	Involving physical pain, energy, sleep, etc.
Psychological field	6	Includes emotional, cognitive, and self-esteem
Social relations area	3	Focus on relationships, social support, etc.
Environmental field	10	Covers living environment, transportation, economic situation, etc.

### 5.2 Testing procedure

During the experiment, data collection was conducted strictly according to the predefined schedule and methods to ensure accuracy and completeness. For the experimental group, health data collected via smart devices were regularly organized and backed up by professional technicians. Data from smart wristbands and intelligent health monitoring devices were collected at fixed times each day, initially screened to remove anomalies, and then processed.

Following data collection, data cleaning was performed to eliminate duplicates, outliers, and missing values. Subsequently, data preprocessing techniques such as standardization and normalization were applied to enhance data quality. For questionnaire responses and interview data, coding and entry were conducted immediately after collection. A data validation mechanism was implemented during data entry, involving multiple rounds of verification to ensure accuracy. Throughout the entire testing process, trained professionals assisted and guided participants, informing them of the study’s purpose and significance.

### 5.3 Data analysis

Statistical analyses were conducted using IBM SPSS Statistics 25.0. First, descriptive statistics were used to summarize key indicators for both groups of older adult participants.

To assess baseline comparability, independent sample t-tests were performed on pre-test measurements. If no significant differences were detected, comparability between groups was confirmed. Next, paired sample *t*-tests were employed to examine within-group changes before and after the intervention. Additionally, analysis of variance (ANOVA) was used to determine whether differences in changes between the experimental and control groups were statistically significant.

The significance level was set at *α* = 0.05, with *p* < 0.05 indicating statistically significant differences. The independent sample t-test assumed data followed a normal distribution. Homogeneity of variance was tested using Levene’s test; if variances were equal, the Student’s *t*-test was applied, whereas the Welch *t*-test was used for unequal variances. These rigorous statistical methods ensured the scientific validity and reproducibility of the analysis.

### 5.4 Evaluation and measurement indicator system construction

#### 5.4.1 Principles for selecting evaluation indicators

To comprehensively and objectively evaluate the “SMT” integrated health system, the selection of evaluation indicators followed the principles of scientific accuracy, systematics, operability, and representativeness. Scientific Accuracy: This principle requires that the indicators accurately reflect the essential characteristics and operational principles of the system, based on scientific theories and methods. Systematic Approach: The indicator system must encompass all aspects of the system and form an integrated whole, providing an evaluation from various dimensions. Operability: The indicators should be easy to obtain and measure, ensuring feasibility in practical applications. Representativeness: The selected indicators must highlight the system’s key elements and core functions, ensuring they are highly representative of the system. All indicators were selected with reference to relevant domestic and international studies and standards to ensure their scientific validity and authority.

#### 5.4.2 Specific evaluation indicators

Based on the above principles, an evaluation indicator system was constructed, which includes four primary categories: system functions, service quality, user experience, and social impact. Each primary category is subdivided into several secondary indicators (Table 5).

**Table 5:** Measurement and evaluation index system of “Sport, Medicine and Tourism” intelligent integrated recreation system.

Primary indicator	Secondary indicator	Tertiary indicator
System function (A)	Data collection accuracy (A1)	The degree of agreement between the health data collected by the smart device and the data measured by the professional medical equipment is measured by comparing and analyzing the consistency of the two data
	Data analysis and processing capabilities (A2)	The speed and accuracy of the system’s analysis of large amounts of health data, behavioral data, etc., and whether it can mine valuable information, e.g., whether it can predict health risks based on the data, etc.
	Accuracy of service recommendations (A3)	Match between physical exercise programs, medical services, and tourism activities recommended by the system based on the needs and data of the older adult and the actual needs of the older adult, assessed through user feedback and actual results
Quality of service (B)	Specialization of medical services (B1)	The professional qualifications, clinical experience, and accuracy of diagnosis and recommendations of online medical consulting doctors can be assessed by the doctor’s education, years of practice, and patient evaluations
	Suitability of sports services (B2)	Whether the physical exercise program is in line with the physical condition and exercise capacity of the older adult, and whether the exercise instruction is professional, with professionals assessing the reasonableness of the exercise program and the professionalism of the instruction
	Security of tourism services (B3)	Whether measures are in place to safeguard the safety of the older adult in terms of the itinerary of tourism activities, transportation, accommodation, etc., in terms of the provision of safety facilities and the assessment of the risks of the itinerary
	Comfort in tourism services (B4)	Whether meals, rest facilities, etc., during tourism meet the comfort needs of the older adult, as assessed through satisfaction surveys of the older adult
User experience (C)	Ease of operation (C1)	Whether the interface design of the system APP is simple and easy to understand, whether the operation process is convenient and fast, and the difficulty for the older adult to learn how to use it, which will be evaluated through user operation test and feedback.
	Degree of personalization (C2)	The extent to which the services provided by the system meet the individualized needs of older persons and the degree of customization of the services, assessed on the basis of the customization options of the services and the extent to which they meet individualized needs
	Satisfaction (C3)	Older adult people’s satisfaction with the overall services of the “Sport-Medicine-Tourism” Wisdom Integration Recreation System was obtained through a questionnaire survey, which included satisfaction evaluations of various aspects of the services.
	Promoting the development of the older adult care industry (D1)	The system’s role in promoting the local older adult care industry in terms of economic growth and optimization of industrial structure, as measured by indicators such as growth in industrial scale and increase in the number of employed people
	Promotion of social cohesion (D2)	Positive impact of the system on the social integration of older persons, promotion of intergenerational exchanges and enhancement of community cohesion, assessed in terms of increased socialization of older persons and increased participation in community activities

#### 5.4.3 Evaluation methods

The evaluation of the “SMT” integrated health system uses the Analytic Hierarchy Process (AHP) to determine the weight of each evaluation indicator. A panel of 10 experts, scholars, and industry practitioners in the field of older adult care was invited to conduct pairwise comparisons based on the importance of each indicator and construct a judgment matrix. Using specialized software, the maximum eigenvalue of the judgment matrix and its corresponding eigenvector are calculated. The eigenvector is then normalized to obtain the relative weights of each indicator. Consistency tests are conducted to ensure the rationality of the weights. For instance, when constructing the judgment matrix, experts use their experience and expertise to conduct pairwise comparisons of the primary indicators (e.g., system functions, service quality) and the corresponding secondary indicators. They assess which indicators are more important and to what extent. The resulting weights reflect the relative importance of each indicator in the evaluation system. Next, the Fuzzy Comprehensive Evaluation Method is used for a comprehensive evaluation of the “SMT” integrated health system. The evaluation levels are divided into five categories: “Excellent,” “Good,” “Moderate,” “Poor,” and “Very Poor,” corresponding to score ranges [90, 100], [75, 89], [60, 74], [45, 59], and [0, 44], respectively. For each secondary indicator, data from surveys and field research are used to perform single-factor evaluations to determine how much each indicator belongs to each evaluation level, forming a fuzzy relationship matrix. For example, for the secondary indicator “Ease of Use,” the survey results are analyzed to determine the proportion of older adult participants who rated the system as “Excellent,” “Good,” “Moderate,” “Poor,” or “Very Poor.” This information is used to determine the values in the fuzzy relationship matrix. Finally, the weights of the indicators are combined with the fuzzy relationship matrix through a synthesis operation to obtain the overall evaluation result for the “SMT” integrated health system. By using fuzzy comprehensive evaluation, the influence of multiple evaluation indicators is considered, leading to a more comprehensive and objective result. In the Analytic Hierarchy Process, the specific steps and formulas for constructing the judgment matrix, calculating the eigenvector, and performing consistency checks are detailed to ensure the scientific validity and reproducibility of the results.

### 5.5 Experimental results and evaluation conclusions

#### 5.5.1 Experimental results

After a 12-month experiment, the data analysis results show that, in terms of physical health indicators, the experimental group of older adult individuals exhibited significant improvements. Specifically, their blood pressure, blood sugar, and blood lipid levels were effectively controlled, while their cardiovascular function and physical activity ability saw marked enhancement. When compared to the control group, the differences were statistically significant (*p* < 0.05). Detailed data can be found in Table 6.

**Table 6:** Comparison of changes in physical health indicators between the experimental and control groups.

Norm	Experimental group (pre-test)	Experimental group (post-test)	Control group (pre-test)	Control group (post-test)	*P*-value
Systolic blood pressure (mmHg)	130.5 ± 10.2	122.3 ± 8.5	129.8 ± 9.8	128.6 ± 9.5	<0.05
Diastolic blood pressure (mmHg)	82.4 ± 6.5	78.2 ± 5.8	81.9 ± 6.2	81.0 ± 6.0	<0.05
Fasting blood glucose (mmol/L)	6.2 ± 0.8	5.6 ± 0.6	6.1 ± 0.7	6.0 ± 0.7	<0.05
2-h postprandial blood glucose (mmol/L)	8.5 ± 1.2	7.8 ± 1.0	8.4 ± 1.1	8.3 ± 1.0	<0.05
Total cholesterol (mmol/L)	5.5 ± 0.6	5.1 ± 0.5	5.4 ± 0.5	5.3 ± 0.5	<0.05
Triglycerides (mmol/L)	1.8 ± 0.4	1.5 ± 0.3	1.7 ± 0.3	1.6 ± 0.3	<0.05
LDL cholesterol (mmol/L)	3.6 ± 0.5	3.2 ± 0.4	3.5 ± 0.4	3.4 ± 0.4	<0.05
HDL cholesterol (mmol/L)	1.1 ± 0.2	1.3 ± 0.2	1.1 ± 0.2	1.2 ± 0.2	<0.05
Lung capacity (ml)	2,200 ± 300	2,400 ± 350	2,150 ± 280	2,200 ± 300	<0.05
Maximum oxygen uptake (ml/kg/min)	18.5 ± 3.0	20.0 ± 3.5	18.0 ± 2.8	18.5 ± 3.0	<0.05
ADL Scale Score	75 ± 10	82 ± 8	76 ± 9	78 ± 9	<0.05

In terms of psychological health indicators, the experimental group of older adult individuals showed a significant reduction in their depression scale scores and a significant increase in their life satisfaction scale scores. When compared to the control group, the differences were statistically significant (*p* < 0.05). Detailed data can be found in Table 7.

**Table 7:** Comparison of changes in mental health indicators between the experimental and control groups.

Norm	Experimental group (pre-test)	Experimental group (post-test)	Control group (pre-test)	Control group (post-test)	*P*-value
GDS-15 scale score	8.2 ± 2.5	5.5 ± 1.8	8.0 ± 2.3	7.8 ± 2.2	<0.05
SWLS scale score	4.0 ± 1.0	5.5 ± 1.2	4.1 ± 1.1	4.3 ± 1.0	<0.05

In terms of social participation indicators, the experimental group of older adult individuals showed a significant increase in the number of community activities they participated in, as well as a larger number of interest groups joined and a bigger social circle compared to the control group. The differences were statistically significant (*p* < 0.05). Detailed data can be found in Table 8.

**Table 8:** Comparison of changes in social participation indicators between the experimental and control groups.

Norm	Experimental group (pre-test)	Experimental group (post-test)	Control group (pre-test)	Control group (post-test)	*P*-value
Participation in community activities (times/month)	2.5 ± 1.0	4.8 ± 1.5	2.3 ± 0.9	2.8 ± 1.2	<0.05
Number of interest groups joined (number)	1.2 ± 0.5	2.0 ± 0.8	1.1 ± 0.4	1.3 ± 0.6	<0.05
Social circle size (persons)	10.5 ± 3.0	13.8 ± 3.5	10.2 ± 2.8	11.0 ± 3.0	<0.05

In terms of quality of life indicators, the experimental group scored significantly higher than the control group in the physical, psychological, social relationships, and environmental domains, with the differences being statistically significant (*p* < 0.05). Detailed data can be found in Table 9.

**Table 9:** Comparison of changes in quality of life indicators between the experimental and control groups.

Norm	Experimental group (pre-test)	Experimental group (post-test)	Control group (pre-test)	Control group (post-test)	*P*-value
Physiological domain score	3.2 ± 0.5	3.8 ± 0.6	3.1 ± 0.5	3.3 ± 0.5	<0.05
Psychological domain score	3.0 ± 0.6	3.6 ± 0.7	3.0 ± 0.5	3.2 ± 0.6	<0.05
Score in the area of social relations	3.3 ± 0.4	3.8 ± 0.5	3.2 ± 0.4	3.4 ± 0.5	<0.05
Score in the area of environment	3.1 ± 0.5	3.7 ± 0.6	3.0 ± 0.5	3.2 ± 0.5	<0.05

#### 5.5.2 Evaluation conclusion

The evaluation of the “SMT” intelligent integrated older adult care system, using the AHP and fuzzy comprehensive evaluation method, shows the following results:

- System Function: The data collection accuracy is high, data analysis and processing capabilities are strong, and service recommendations are relatively precise.
- Service Quality: The professionalism of medical services, suitability of sports services, and safety and comfort of tourism services are adequately ensured.
- User Experience: The ease of operation and personalization of services are well recognized by the older adult users, with high overall satisfaction.
- Social Impact: The system has a positive role in promoting the development of the older adult care industry and contributing to social harmony.

The comprehensive evaluation result is rated as “Good,” but there is still room for improvement in certain system functions and the comfort of tourism services. The specific evaluation scores and ratings are shown in Table 10.

**Table 10:** Comprehensive evaluation results of the “Sport, Medicine and Tourism” intelligent integration recreation system.

Primary indicator	Score	Rating levels
System function	80	Favorable
Quality of Service	78	Favorable
User experience	82	Favorable
Social impact	76	Favorable
Synthesized assessment	79	Favorable

In conclusion, the “SMT” intelligent integrated older adult care system has demonstrated significant effects in improving the physical and mental health of the older adult, promoting social participation, and enhancing quality of life. It performs well in system functions, service quality, user experience, and social impact. However, it still requires optimization and improvement in response to the issues identified during the evaluation in order to further enhance the system’s operational effectiveness and service quality.

The system’s strengths include high data collection accuracy and good user experience, while areas for improvement include the comfort of tourism services. It is recommended to optimize the dining and rest facilities within the tourism services to further improve the overall performance of the system.

## 6 Conclusions and discussion

### 6.1 Conclusion

An intelligent integrated older adult care system was conceptualized and operationalized in this study through the lens of Science and Technology Studies (STS) and Actor-Network Theory. Designed to address the nuanced and heterogeneous needs of older adults at the micro level, the system consolidated cross-sector resources from sports, medicine, and tourism, and was structured around a data-driven, technology-enabled, and service-synergized architecture. A longitudinal field intervention, implemented over a 12-month period in Jinan, Qingdao, and Rizhao, provided empirical evidence supporting the system’s efficacy in enhancing physical health, psychological well-being, social engagement, and perceived quality of life among participants.

Relative to existing “Sports-Medicine-Wellness” models, the proposed framework demonstrated superior performance in service heterogeneity, adaptive responsiveness, and user-centered satisfaction. It simultaneously addressed structural limitations commonly reported in prior studies, including inadequate cross-domain integration, superficial technological application, and fragmented service delivery (29, 30). These findings further underscore that older adult care systems, when anchored in user-centric design and informed by interdisciplinary thinking at the levels of system architecture, operational dynamics, and supply chain design, can achieve greater comprehensiveness and inclusivity (31–33).

### 6.2 Discussion

While considerable attention has been given to subfields such as Sports-Medicine Wellness, Integrated Medical and older adult Care, and Smart Health, prior studies have largely remained focused on isolated subsystem optimization—for instance, the development of smart fitness devices (34), integration of telemedicine platforms (35), or the linear consolidation of wellness tourism offerings (36). In contrast, the present study advances a ternary integration framework that systematically bridges sports, medicine, and tourism. It introduces an operational model structured around a closed-loop process comprising micro-need identification, service chain reconfiguration, and collaborative feedback—an approach that constitutes a novel contribution to current discourse.

In divergence from the “multi-module parallel collaboration” model introduced by Sakaguchi-Tang et al. (37), the system developed here prioritizes dynamic adaptability and proactive user participation. On the one hand, service generation is achieved through real-time collection of behavioral and health data via smart devices, followed by AI-powered personalized recommendations. This mechanism surpasses the passive response architecture typical of static information systems. On the other hand, a four-dimensional interaction framework—incorporating people, objects, data, and environments—was embedded to support the transition from siloed service provision to a fully networked delivery system. Further, the system was found to enhance digital autonomy among older users, thereby addressing long-standing concerns in the literature. As highlighted by Yazdani-Darki, technological usability remains a major barrier, often resulting in “technology islands” for older adults. By adopting age-adaptive interface design and implementing targeted skill training programs, the present system significantly improved both digital literacy and engagement willingness. These findings underscore the importance of shifting from mere functional deployment to genuinely human-centered intelligent care solutions.

Nevertheless, several limitations should be acknowledged. First, the study sample was drawn primarily from urban areas in eastern China, limiting generalizability to rural and remote populations. Second, the one-year duration of the intervention precludes robust conclusions regarding long-term impact and system sustainability. Third, the tourism service component remains suboptimal in terms of comfort and cultural adaptability. These limitations align with observations by Hu et al. (38), who emphasized the necessity of personalized experience design in wellness tourism, suggesting that future system refinement should target cultural integration, ecological compatibility, and access optimization.

Subsequent research efforts should proceed along three major trajectories: expanding geographic and demographic scope to test adaptability across socioeconomic contexts; conducting longitudinal studies to capture sustained effects on chronic illness management and psychological resilience; and integrating stakeholder perspectives—including caregivers, family members, and policymakers—to construct a multidimensional assessment framework that can inform both technical refinement and policy innovation.

## Data Availability

The original contributions presented in the study are included in the article/supplementary material, further inquiries can be directed to the corresponding author.
